# Prognostic value of the neutrophil percentage-to-albumin ratio for mortality in ICU patients with myocardial infarction: a retrospective cohort and machine learning analysis

**DOI:** 10.3389/fcvm.2025.1631493

**Published:** 2025-12-19

**Authors:** Zhantao Cao, Zhonghui Lin, Xuejing Xu, Zhanglu Zhang, Xuanjing Chen, Jun Chen, Yunsu Wang

**Affiliations:** Department of Cardiology, Xiamen TCM Hospital Affiliated to Fujian University of Traditional Chinese Medicine, Xiamen, Fujian, China

**Keywords:** neutrophil percentage-to-albumin ratio, myocardial infarction, all-causemortality, machine learning, MIMIC-IV database

## Abstract

**Background:**

Although the neutrophil percentage-to-albumin ratio (NPAR) has shown prognostic value in multiple clinical conditions, its prognostic accuracy for myocardial infarction (MI) patients receiving intensive care has yet to be clearly defined. To our knowledge, this study is the first to comprehensively evaluate the prognostic role of NPAR in ICU-admitted MI patients, integrating both conventional Cox regression and machine learning approaches to address an existing gap between general MI cohorts and critically ill populations.

**Method:**

Using data from the MIMIC-IV v3.1 database, we retrospectively included 1,759 ICU-admitted MI patients and calculated NPAR at admission. Primary and secondary outcomes were 30-day and 360-day all-cause mortality, respectively. Kaplan–Meier curves and log-rank tests compared survival across tertiles. Multivariate Cox models assessed associations, with restricted cubic splines evaluating nonlinearity. Machine learning models incorporating NPAR were developed to predict 30-day mortality, and model performance was assessed using the area under the receiver operating characteristic curve (AUC).

**Result:**

The 30-day and 360-day all-cause mortality rates were 24% and 38%, respectively. Kaplan–Meier analysis revealed significantly lower survival probabilities in patients with higher NPAR levels. Adjusted Cox regression showed that those in the highest NPAR tertile had an increased risk of 30-day (HR: 2.03, 95% CI: 1.51–2.73, *p* < 0.001) and 360-day (HR: 1.81, 95% CI: 1.45–2.26, *p* < 0.001) mortality. Machine learning models incorporating NPAR achieved an AUC of up to 0.81 for predicting 30-day death.

**Conclusion:**

The NPAR serves as an independent predictor of mortality at 30 and 360 days in MI patients admitted to the intensive care unit (ICU). When integrated into machine learning models, NPAR enhances predictive accuracy. These results indicate that NPAR serves as an independent predictor of short- and long-term mortality in ICU-admitted MI patients. Given its simplicity and accessibility from routine laboratory tests, NPAR can be feasibly incorporated into clinical decision-making and risk stratification protocols in critical care settings to facilitate individualized risk assessment and improve outcomes.

## 1 Introduction

Myocardial infarction (MI) remains a leading cause of morbidity and mortality worldwide (1). According to the 2025 American Heart Association (AHA) global update, approximately 254 million people were living with ischemic heart disease (IHD) in 2021, and nearly 9 million deaths were attributed to IHD globally (2). As a major component of IHD, MI continues to impose a profound public-health burden. A 2023 systematic review estimated that the global prevalence of MI was approximately 1.72%, with higher rates among elderly and male populations (3). Furthermore, patients with MI frequently require intensive care due to severe complications such as cardiogenic shock, arrhythmias, or respiratory failure. For instance, findings from a large cohort investigation revealed that 62% of patients hospitalized for acute myocardial infarction (AMI) required intensive care unit (ICU) care, with about 5% of these critically ill individuals dying within 30 days (4). The development and advancement of MI are strongly influenced by underlying inflammatory processes. For instance, inflammatory responses contribute to the destabilization of atherosclerotic plaques and promote thrombus formation, with elevated inflammatory biomarkers correlating with poorer MI outcomes (5). After MI, inflammation triggers the mobilization of neutrophils and other immune components to the damaged myocardial tissue, which may worsen cardiac injury and affect tissue repair mechanisms (6). Effective prognostic stratification is crucial, as it allows clinicians to identify high-risk individuals who may benefit from more intensive monitoring or tailored therapeutic interventions. Therefore, inflammatory biomarkers may serve as important predictors of prognosis in patients with MI (7).

The neutrophil percentage-to-albumin ratio (NPAR) is an inflammatory biomarker derived from the ratio of neutrophil percentage to serum albumin level. Previous studies have shown that neutrophils exacerbate inflammation and tissue injury following MI by releasing proteases and reactive oxygen species (8). Similarly, hypoalbuminemia correlates with adverse outcomes in MI patients, potentially due to reduced albumin synthesis caused by inflammation or increased capillary leakage (9). In this context, neutrophils function as mediators of inflammation, while albumin simultaneously reflects inflammatory and nutritional status. Therefore, NPAR may provide an integrated assessment of systemic inflammation and overall physiological status in MI patients. Unlike conventional biomarkers such as CRP, NLR, or albumin alone, NPAR simultaneously reflects both inflammatory activation and nutritional depletion, thereby capturing the complex inflammatory–metabolic interplay that characterizes critically ill states. This dual-dimensional nature may render NPAR more sensitive and comprehensive in prognostic evaluation compared with single inflammatory or nutritional indicators. Prior studies have established the prognostic value of the NPAR in specific high-risk cardiovascular cohorts, including those with ST-segment elevation MI, cardiogenic shock, atrial fibrillation, or chronic total occlusion (10–13). However, its utility has not been systematically examined in the broader, unselected population of critically ill MI patients using large-scale datasets and advanced modeling. Accordingly, the primary objective of this study was to investigate the association between NPAR and both short- and long-term all-cause mortality in a large cohort of critically ill MI patients and to validate its predictive power using machine learning models.

## 2 Methods

### 2.1 Data source

This study utilized patient data obtained from MIMIC-IV (v3.1). The MIMIC-IV database contains detailed, de-identified clinical information from patients admitted to the ICU at Beth Israel Deaconess Medical Center, covering the period from 2008 to 2022. The database contains rich patient-level data, including demographic details, diagnostic classifications, vital parameters, laboratory test results, medication records, and discharge status (14). One of the contributing researchers, Zhantao Cao, fulfilled the requirements of the Collaborative Institutional Training Initiative ethical certification and was granted authorized access to the MIMIC-IV dataset (Certification Number: 14336451). All data extraction and handling procedures strictly adhered to database usage guidelines and ethical standards.

### 2.2 Participants

**Figure 1:**
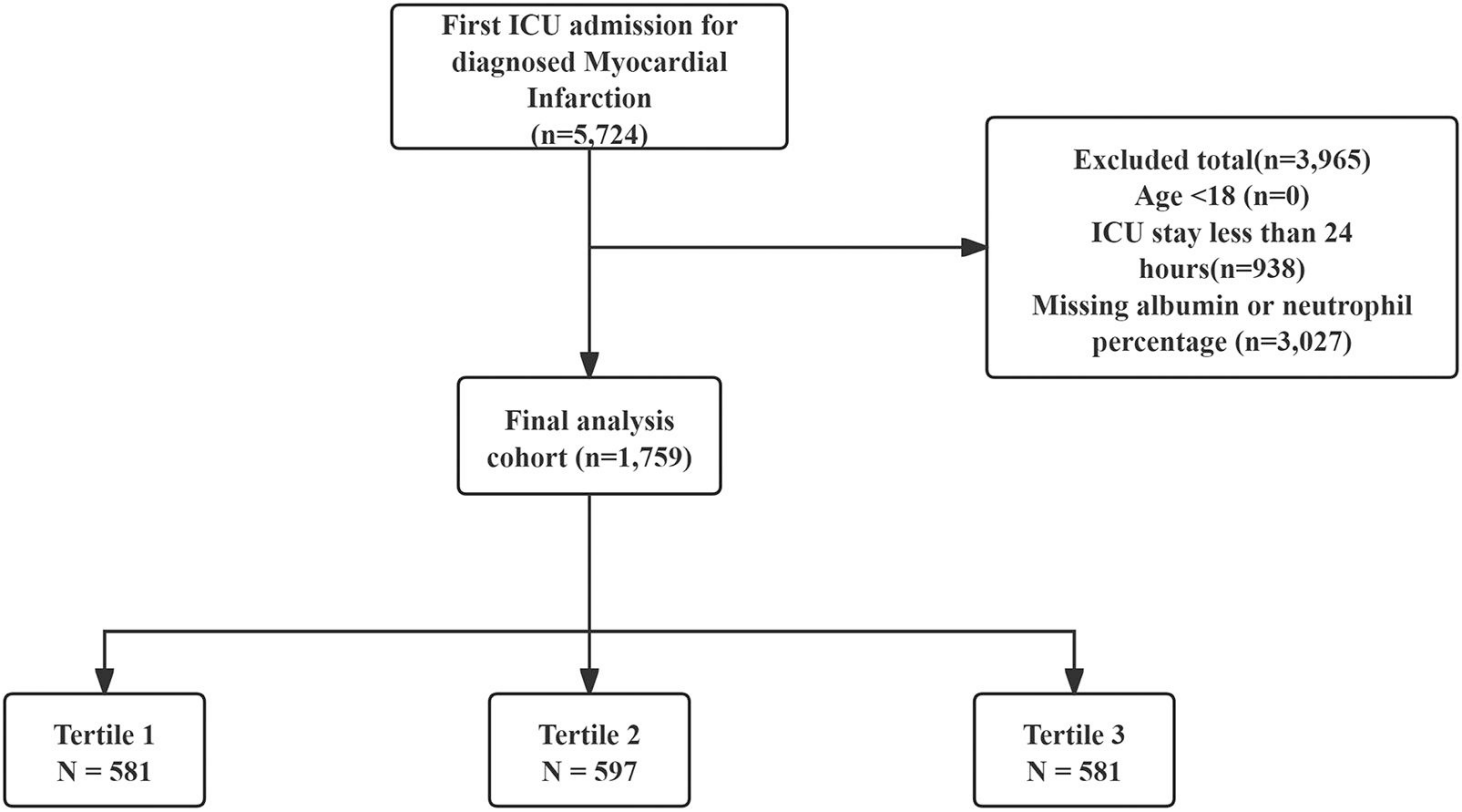
Patient screening flow from the MIMIC database.

Patients were included if their initial ICU admission was associated with a confirmed diagnosis of MI, identified using ICD-9 or ICD-10 diagnostic codes. Exclusion conditions included: (1) Individuals admitted to the ICU before reaching the age of 18; and (2) ICU durations of less than 24 h; and (3) missing critical laboratory data (neutrophil percentage or serum albumin). In total, 1,759 qualified patients were eventually enrolled and categorized into three groups according to the tertile distribution of their NPAR levels. The tertile cut-off values were determined based on the distribution percentiles of NPAR within the current cohort to ensure balanced subgroup sizes. The detailed patient selection process is illustrated in Figure 1.

### 2.3 Data collection

Data retrieval from the MIMIC-IV database was performed using Structured Query Language (SQL) through the pgAdmin interface (version 4). All variables were obtained from clinical records documented during the initial 24 h period after ICU admission. Specifically, the following seven categories of information were obtained: demographics, vital parameters, underlying comorbid conditions, laboratory indicators, medication usage, clinical interventions, and severity scores. A complete list of all variables is provided in Supplementary Table S1.The value of NPAR was calculated based on the following formula: NPAR = Neutrophil percentage (%) × 100/Albumin (g/dL) (15).

To handle missing data while minimizing potential bias, we first excluded variables with a missingness rate exceeding 20% (Supplementary Table S2). For the remaining variables with less than 20% missing data, we performed multiple imputations by chained equations (MICE) using the “mice” package in R, assuming the data were missing at random (MAR). We generated five complete datasets (m = 5), employing predictive mean matching (PMM) for continuous variables and logistic regression for binary categorical variables. All subsequent analyses were performed on each of the five datasets, and the results were pooled using Rubin's rules to generate the final estimates and confidence intervals.

### 2.4 Statistical analysis

Continuous variables were expressed as mean ± SD or median with IQR, depending on normality, which was assessed using the Kolmogorov–Smirnov test. Categorical data were summarized as counts and percentages. Between-group comparisons used *t*-tests or one-way analysis of variance for normally distributed variables, and Mann–Whitney *U* or Kruskal–Wallis tests for skewed data.

Kaplan–Meier survival curves were generated to illustrate mortality patterns across NPAR groups, and the log-rank test was applied to evaluate statistical differences between them. In addition, univariate Cox regression was utilized to initially screen variables potentially linked to all-cause mortality. Multicollinearity among all potential covariates considered for the study was assessed using the Variance Inflation Factor (VIF). A VIF value > 5 was considered indicative of significant multicollinearity.

To evaluate the relationship between NPAR and all-cause mortality, Cox proportional hazards regression was conducted to calculate hazard ratios (HRs) and their 95% confidence intervals (CIs), with appropriate adjustment for potential confounders. The selection of covariates for the final multivariable models was guided by a strict criterion: we included only those variables that were both statistically significant in the univariate analysis and confirmed to be clinically relevant based on physician judgment. Model 1 was a crude model without any covariate adjustment. Model 2 adjusted for core demographic factors, namely age, gender, and race. Model 3 further adjusted for additional adjustments for clinical and laboratory variables such as heart rate, systolic blood pressure(SBP), pulse oxygen saturation(SpO2),congestive heart failure (CHF), atrial fibrillation (AF), red cell distribution width(RDW), blood urea nitrogen (BUN), partial thromboplastin time (PT), urine output, and Beta-blocker use. The proportional hazards (PH) assumption for the final multivariable Cox models was tested using statistical tests based on the scaled Schoenfeld residuals. A *p*-value > 0.05 from the global test was considered to indicate no violation of the assumption.

To assess potential nonlinear associations between NPAR and mortality, we performed restricted cubic spline (RCS) regression. In accordance with established biostatistical guidelines and common practice in cardiovascular literature (16), we specified three knots placed at the 10th, 50th, and 90th percentiles of the NPAR distribution.

To evaluate the stability of NPAR as a prognostic indicator for all-cause mortality, subgroup analyses were performed within strata defined by various baseline characteristics: age, CHF, AF, diabetes, hypertension, and Beta-blocker use. To evaluate potential interactions between NPAR and individual stratification variables, the likelihood ratio test was performed. Statistical significance was defined as a two-sided *p*-value less than 0.05. All analyses were performed using R (version 4.4.2).

### 2.5 Construction and assessment of the prognostic models

The Boruta method is a robust variable selection technique built upon the principles of the random forest algorithm, aimed at detecting all predictors within a dataset that are significantly associated with the target outcome (17). It operates by creating “shadow features”—randomly permuted versions of the original variables—and then evaluates the significance of each actual feature by comparing its importance against these shadow counterparts. Feature importance is quantified using the Z-score derived from the random forest model. A feature is deemed “important” (marked in green) and retained if it consistently achieves a Z-score notably greater than the highest Z-score of all shadow features over multiple iterations. In contrast, variables that perform worse than the shadow features are considered “unimportant” (marked in red) and excluded. Features identified as important are regarded as contributing significantly to the model's predictive capability. In the present study, the Boruta algorithm was applied to determine the predictive importance of NPAR among the candidate variables.

The identified features were then integrated into several machine learning (ML) algorithms for model construction. The dataset was randomly partitioned into a training set (70%) and a testing set (30%) using a stratified sampling approach to ensure a consistent proportion of mortality events in both subsets. Within the training set, we performed hyperparameter optimization for each of the six machine learning algorithms: Decision tree (DT), ridge regression classifier (Ridge), random forest (RF), extreme gradient boosting (XGBoost), multilayer perceptron (MLP), and elastic net regression (ENet). This was achieved using a five-fold cross-validation strategy coupled with a grid search to identify the combination of hyperparameters that yielded the highest average AUC. The final models were then trained on the entire training dataset using these optimized parameters and subsequently evaluated for performance on the independent testing set. Model performance was comprehensively assessed for discrimination using the area under the receiver operating characteristic curve (AUC-ROC), for calibration using calibration plots, and for clinical utility using decision curve analysis.

## 3 Results

### 3.1 Baseline characteristics

This study enrolled 1,759 patients diagnosed with MI admitted to the ICU. Participants had a median age of 70.67 years (interquartile range: 62.01–79.60), and males accounted for 65% of the study population. The overall median NPAR value was 23.82, with an interquartile range of 20.26 to 28.86. All-cause mortality was 24% at 30 days and increased to 38% by 360 days (Table 1).

**Table 1:** Characteristics and outcomes of participants categorized by NPAR.

Characteristic	Overall *N* = 1,759	T1 *N* = 581	T2 *N* = 597	T3 *N* = 581	*p*-value
Age (year)	70.67 (62.01, 79.60)	68.88 (60.38, 76.79)	71.61 (63.26, 79.17)	71.59 (63.27, 81.89)	<0.001
Gender, *n* (%)					<0.001
Female	621 (35%)	165 (28%)	222 (37%)	234 (40%)	
Male	1,138 (65%)	416 (72%)	375 (63%)	347 (60%)	
Race, *n* (%)					0.657
Other	654 (37%)	208 (36%)	223 (37%)	223 (38%)	
White	1,105 (63%)	373 (64%)	374 (63%)	358 (62%)	
BMI	27.90 (24.38, 32.25)	27.83 (24.84, 32.42)	28.00 (24.17, 32.23)	27.72 (23.95, 32.19)	0.463
Heart rate (bmp)	86.00 (76.00, 101.00)	82.00 (75.00, 92.00)	86.00 (75.00, 99.00)	94.00 (80.00, 109.00)	<0.001
SBP (mmHg)	118.00 (104.00, 134.00)	118.00 (103.00, 133.00)	119.00 (105.00, 134.00)	116.00 (102.00, 134.00)	0.343
DBP (mmHg)	66.00 (56.00, 78.00)	65.00 (55.00, 77.00)	67.00 (57.00, 78.00)	66.00 (54.00, 79.00)	0.428
RR (bmp)	19.00 (16.00, 24.00)	18.00 (15.00, 22.00)	20.00 (16.00, 24.00)	21.00 (17.00, 26.00)	<0.001
Temperature (°C)	36.67 (36.39, 37.00)	36.67 (36.39, 36.94)	36.67 (36.40, 36.94)	36.70 (36.40, 37.00)	0.409
Spo2 (%)	98.00 (95.00, 100.00)	98.00 (96.00, 100.00)	97.00 (95.00, 100.00)	97.00 (94.00, 100.00)	<0.001
SOFA	2.00 (0.00, 4.00)	2.00 (0.00, 4.00)	2.00 (0.00, 4.00)	2.00 (0.00, 4.00)	0.055
OASIS	34.00 (28.00, 41.00)	31.00 (25.00, 38.00)	33.00 (28.00, 39.00)	38.00 (32.00, 44.00)	<0.001
CCI	6.00 (4.00, 9.00)	5.00 (4.00, 8.00)	6.00 (5.00, 9.00)	7.00 (5.00, 9.00)	<0.001
CHF, *n* (%)	941 (53%)	233 (40%)	369 (62%)	339 (58%)	<0.001
CeVD *n* (%)	286 (16%)	85 (15%)	94 (16%)	107 (18%)	0.198
COPD, *n* (%)	436 (25%)	115 (20%)	153 (26%)	168 (29%)	0.001
AF, *n* (%)	700 (40%)	200 (34%)	240 (40%)	260 (45%)	0.002
AKI, *n* (%)	1,493 (85%)	456 (78%)	518 (87%)	519 (89%)	<0.001
Sepsis, *n* (%)	1,069 (61%)	239 (41%)	347 (58%)	483 (83%)	<0.001
Diabetes, *n* (%)	746 (42%)	251 (43%)	256 (43%)	239 (41%)	0.745
Hypertension, *n* (%)	1,340 (76%)	457 (79%)	461 (77%)	422 (73%)	0.042
RBC (10^9^/L)	3.55 (2.92, 4.19)	3.65 (2.96, 4.27)	3.71 (2.97, 4.29)	3.34 (2.83, 3.86)	<0.001
WBC (10^9^/L)	12.20 (8.90, 16.90)	10.10 (7.60, 13.50)	12.60 (9.20, 16.50)	15.10 (10.80, 21.10)	<0.001
Platelet(10^9^/L)	188.00 (136.00, 250.00)	170.00 (128.00, 224.00)	198.00 (148.00, 256.00)	196.00 (134.00, 272.00)	<0.001
RDW	14.30 (13.20, 15.90)	13.60 (12.90, 15.00)	14.20 (13.20, 15.60)	15.10 (14.00, 16.90)	<0.001
Neutrophils (%)	80.50 (73.60, 86.00)	72.10 (65.10, 78.37)	81.50 (77.00, 85.40)	85.70 (81.00, 89.90)	<0.001
Albumin (g/dL)	3.30 (2.80, 3.70)	3.85 (3.60, 4.10)	3.40 (3.17, 3.60)	2.70 (2.40, 2.90)	<0.001
NPAR	23.82 (20.26, 28.86)	18.85 (17.26, 20.22)	23.82 (22.60, 25.30)	31.25 (28.92, 35.50)	<0.001
Sodium (mmol/L)	138.00 (135.00, 141.00)	138.00 (136.00, 140.00)	138.00 (135.00, 140.00)	138.00 (134.00, 141.00)	0.830
Potassium (mmol/L)	4.30 (3.90, 4.80)	4.30 (3.90, 4.60)	4.30 (3.90, 4.80)	4.30 (3.80, 4.90)	0.198
Calcium (mg/dL)	8.40 (7.90, 8.90)	8.60 (8.10, 9.10)	8.40 (8.00, 8.90)	8.10 (7.60, 8.60)	<0.001
BUN (mg/dL)	24.00 (16.00, 42.00)	18.00 (13.00, 29.00)	25.00 (17.00, 41.00)	33.00 (20.00, 54.00)	<0.001
Creatinine (mg/dL)	1.20 (0.90, 2.00)	1.00 (0.80, 1.40)	1.20 (0.90, 2.00)	1.50 (1.00, 2.60)	<0.001
INR	1.30 (1.20, 1.60)	1.30 (1.10, 1.50)	1.30 (1.10, 1.60)	1.40 (1.20, 1.70)	<0.001
PT (S)	14.50 (12.60, 17.30)	14.40 (12.40, 16.70)	14.10 (12.50, 17.00)	14.90 (13.20, 18.30)	<0.001
PTT (S)	33.80 (28.20, 55.10)	34.10 (28.40, 56.70)	34.00 (27.90, 59.50)	33.50 (28.30, 47.00)	0.300
Urine output (mL)	1,350.00 (770.00, 2,190.00)	1,685.00 (1,090.00, 2,345.00)	1,359.00 (845.00, 2,175.00)	975.00 (475.00, 1,840.00)	<0.001
Statin, *n* (%)	1,253 (71%)	472 (81%)	459 (77%)	322 (55%)	<0.001
Insulin, *n* (%)	1,180 (67%)	437 (75%)	375 (63%)	368 (63%)	<0.001
Beta-blockers, *n* (%)	1,306 (74%)	481 (83%)	449 (75%)	376 (65%)	<0.001
ACEI/ARB, *n* (%)	529 (30%)	168 (29%)	205 (34%)	156 (27%)	0.015
MV, *n* (%)	1,560 (89%)	520 (90%)	522 (87%)	518 (89%)	0.487
CRRT, *n* (%)	189 (11%)	26 (4%)	55 (9%)	108 (19%)	<0.001
Hospital stay (day)	9.17 (5.92, 16.59)	7.72 (5.79, 11.89)	9.74 (6.01, 16.02)	11.73 (6.38, 21.65)	<0.001
Hospital Mortality (%)	375 (21%)	61 (10%)	107 (18%)	207 (36%)	<0.001
ICU stay (day)	3.13 (1.87, 6.09)	2.28 (1.39, 4.03)	3.17 (1.92, 6.07)	4.23 (2.36, 8.41)	<0.001
ICU Mortality	267 (15%)	39 (7%)	79 (13%)	149 (26%)	<0.001
30-day hospital Mortality (%)	417 (24%)	65 (11%)	122 (20%)	230 (40%)	<0.001
360-day hospital Mortality (%)	662 (38%)	127 (22%)	206 (35%)	329 (57%)	<0.001

NPAR: T1(0.14–21.41), T2(21.41–26.94), T3(26.9–75.00).

BMI, body mass index; SBP, systolic blood pressure; DBP, diastolic blood pressure; RR, respiratory rate; Spo2, pulse oxygen saturation; SOFA, sequential organ failure assessment; OASIS, Oxford Acute Severity of Illness Score; CCI, Charlson Comorbidity Index; CHF, congestive heart failure; CeVD, cerebrovascular disease; COPD, chronic obstructive pulmonary disease; AF, atrial fibrillation; AKI, acute kidney injury; RBC, red blood cell count; WBC, white blood cell count; RDW, red cell distribution width; BUN, blood urea nitrogen; INR, international normalized ratio; PT, prothrombin time; PTT, partial thromboplastin time; NPAR, neutrophil percentage-to-albumin ratio; ACEI/ARB, angiotensin-converting enzyme inhibitor/angiotensin receptor blocker; MV, mechanical ventilation; CRRT, continuous renal replacement therapy.

Based on their NPAR levels at the time of ICU admission, patients were categorized into three groups: T1 (0.14–21.41), T2 (21.41–26.94), and T3 (26.94–75.00). The corresponding median NPAR values were 18.85 (IQR: 17.26–20.22) for T1, 23.82 (IQR: 22.60–25.30) for T2, and 31.25 (IQR: 28.92–35.50) for T3.

Patients in the highest NPAR tertile (T3) were generally older, with higher heart and respiratory rates, and had increased OASIS and CCI scores. They also showed a greater prevalence of AF, AKI, COPD, and sepsis. Laboratory findings revealed lower levels of RBC, platelets, albumin, and calcium, alongside elevated RDW, neutrophils, WBC, BUN, creatinine, INR, PT, and urine output. CRRT use was more frequent in T3, while the use of ACEI/ARB, beta-blockers, and statins was significantly reduced.

With respect to clinical outcomes, 30-day all-cause mortality reached 40% in the T3 group, which was markedly higher than in the T2 (20%) and T1 (11%) groups (*p* < 0.001). Likewise, 360-day mortality was 57% for T3 patients, vs. 35% and 22% in the T2 and T1 groups, respectively (*p* < 0.001).

### 3.2 Clinical outcomes

**Figure 2:**
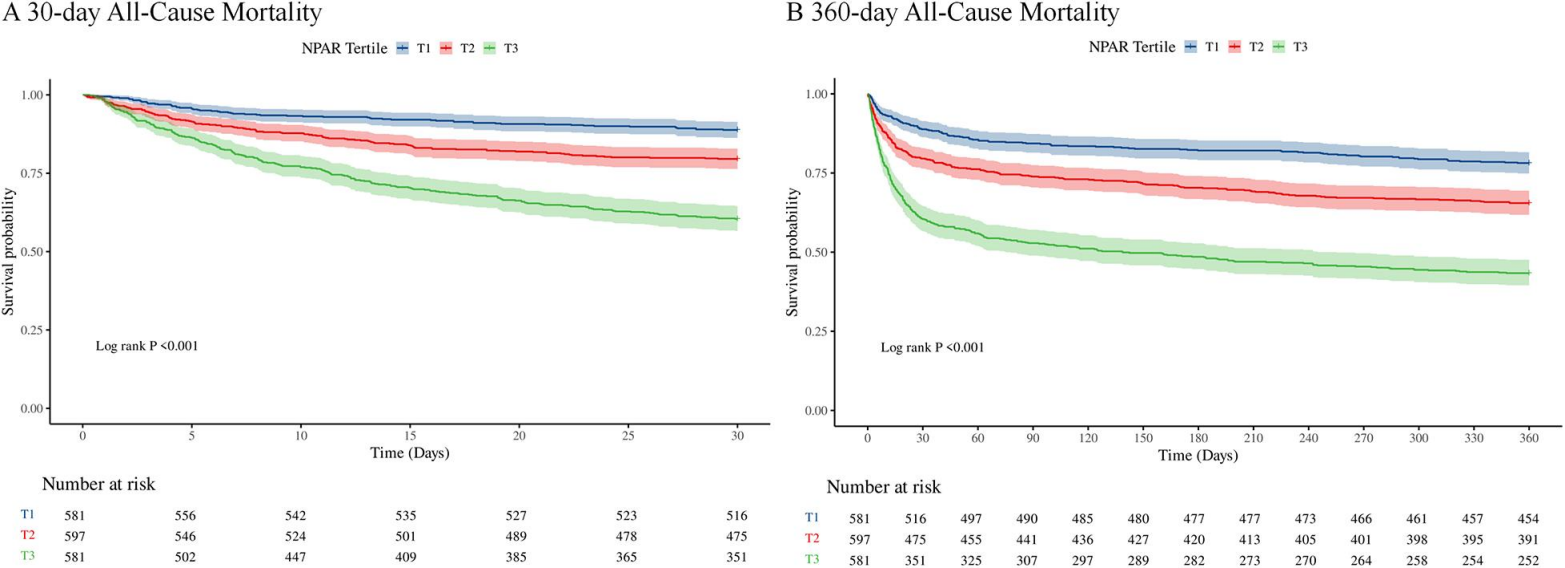
Kaplan–Meier survival curves for 30-day **(A)** and 360-day **(B)** all-cause mortality across NPAR tertiles in ICU myocardial infarction patients.

Figure 2 displays Kaplan–Meier survival plots depicting primary outcome differences among the three NPAR tertile groups. Survival analysis revealed that patients in the highest NPAR tertile had substantially reduced survival probabilities at both 30 days (Figure 2A) and 360 days (Figure 2B), in comparison to those in the lowest tertile. The log-rank test confirmed that these variations reached statistical significance (*p* < 0.001). A comprehensive multicollinearity assessment was performed on all potential covariates. All variables demonstrated Variance Inflation Factor (VIF) values well below 5, indicating that there was no significant multicollinearity among the candidate variables for the final model (Supplementary Table S3).

Supplementary Table S4 displays the results of univariate Cox regression analyses evaluating 30-day all-cause mortality among ICU-admitted patients with MI. Variables yielding *p*-values below 0.05, as well as clinically relevant factors determined by physician judgment, were then incorporated into the multivariate Cox regression model for further analysis. The final multivariable regression model revealed that the following factors were significantly linked to all-cause mortality: age, gender, race, heart rate, SBP, SpO₂, CHF, AF, RDW, BUN, PT, urine output, and Beta-blocker use.

The association between NPAR and all-cause mortality was assessed using multivariable Cox regression, with detailed findings presented in Table 2. Treating NPAR as a continuous variable, its significant association with 30-day mortality persisted across all model adjustments. In the unadjusted model, the HR was 1.07 (95% CI: 1.06–1.08, *p* < 0.001); after partial adjustment, the HR was 1.07 (95% CI: 1.06–1.09, *p* < 0.001); even after full adjustment, the association remained significant, yielding an HR of 1.04 (95% CI: 1.03–1.06, *p* < 0.001). A comparable pattern was noted for 360-day mortality. When NPAR was treated as a tertile-based categorical variable, individuals in the highest tertile (T3) had a markedly higher risk of 30-day mortality compared to those in the lowest tertile (T1). In the unadjusted model (Model 1), the HR was 4.19 (95% CI: 3.18–5.52, *p* < 0.001). Following adjustment for age, gender, and race in Model 2, the HR was modestly reduced to 3.79 (95% CI: 2.87–5.01, *p* < 0.001). In Model 3, after full adjustment, the increased risk persisted with statistical significance, yielding an HR of 2.03 (95% CI: 1.51–2.73, *p* < 0.001). A similar association was identified for 360-day mortality. In every regression model, individuals in the highest NPAR tertile exhibited a substantially greater risk of all-cause mortality at 360 days compared to those in the lowest tertile group. Specifically, the unadjusted model (Model 1) yielded an HR of 3.46 (95% CI: 2.82–4.25, *p* < 0.001); Following adjustment for age, gender, and race in Model 2, the HR was modestly reduced to 3.17 (95% CI: 2.58–3.90, *p* < 0.001); In Model 3, after full adjustment, the increased risk persisted with statistical significance, yielding an HR of 1.81 (95% CI: 1.45–2.26, *p* < 0.001). These findings indicate a potential dose–response effect, where rising NPAR levels are correlated with an increased risk of mortality in ICU patients with MI. The proportional hazards assumption for the final multivariable survival models was formally tested and found to be met. The global Schoenfeld residual tests yielded non-significant *p*-values, confirming that the assumption was not violated for the primary and secondary outcomes (Supplementary Table S5).

**Table 2:** Association between NPAR and 30-day and 360-day all-cause mortality.

Variables	Model1		Model2		Model3	
	HR (95%CI)	*P*	HR (95%CI)	*P*	HR (95%CI)	*P*
30-day Mortality						
NPAR	1.07 (1.06–1.08)	**<**.**001**	1.07 (1.06–1.09)	**<**.**001**	1.04 (1.03–1.06)	**<**.**001**
NPAR Tertile						
T1	1.00 (Reference)		1.00 (Reference)		1.00 (Reference)	
T2	1.94 (1.44–2.63)	**<**.**001**	1.79 (1.32–2.42)	**<**.**001**	1.40 (1.03–1.90)	**0**.**032**
T3	4.19 (3.18–5.52)	**<**.**001**	3.79 (2.87–5.01)	**<**.**001**	2.03 (1.51–2.73)	**<**.**001**
*P* for trend		**<**.**001**		**<**.**001**		**<**.**001**
360-day Mortality						
NPAR	1.07 (1.06–1.08)	**<**.**001**	1.07 (1.06–1.08)	**<**.**001**	1.04 (1.03–1.05)	**<**.**001**
NPAR Tertile						
T1	1.00 (Reference)		1.00 (Reference)		1.00 (Reference)	
T2	1.74 (1.39–2.17)	**<**.**001**	1.61 (1.29–2.01)	**<**.**001**	1.29 (1.03–1.61)	**0**.**029**
T3	3.46 (2.82–4.25)	**<**.**001**	3.17 (2.58–3.90)	**<**.**001**	1.81 (1.45–2.26)	**<**.**001**
*P* for trend		**<**.**001**		**<**.**001**		**<**.**001**

Model1: Crude.

Model2: Adjust: Age, Gender, Race.

Model3: Adjust: Age, Gender, Race, Heart rate, SBP, Spo2, CHF, AF, RDW, BUN, PT, Urine output, Beta-blockers.

HR, hazard ratio; CI, confidence interval; SBP, systolic blood pressure; Spo2, pulse oxygen saturation; CHF, congestive heart failure; AF, atrial fibrillation; RDW, red cell distribution width; BUN, blood urea nitrogen; PT, prothrombin time.

Statistically significant values (*P* < 0.05) are highlighted in bold.

**Figure 3:**
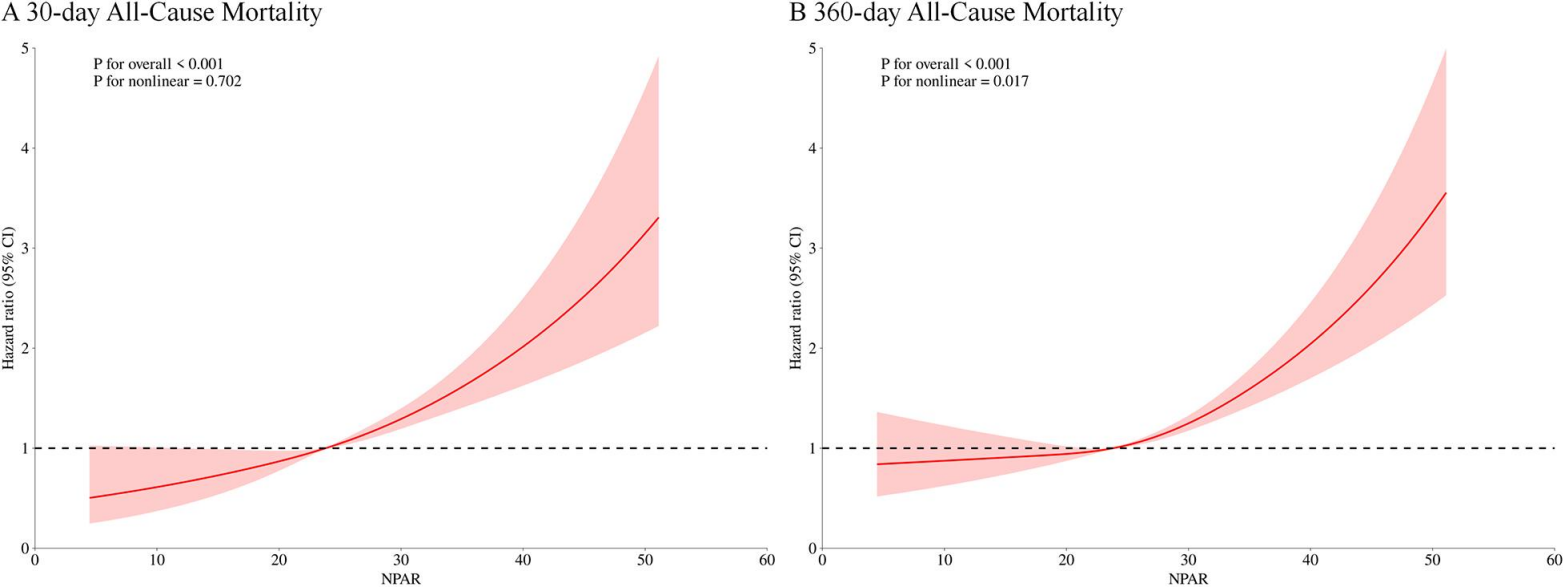
Restricted cubic spline (RCS) analysis of the association between NPAR and all-cause mortality. **(A)** 30-day all-cause mortality; **(B)** 360-day all-cause mortality.

Furthermore, RCS regression demonstrated a significant link between NPAR levels and the risk of all-cause mortality at both 30 and 360 days. For 30-day all-cause mortality, a clear linear trend was observed, with mortality risk rising steadily as NPAR increased (*p* for nonlinearity = 0.702). By contrast, a significant nonlinear relationship between NPAR and 360-day all-cause mortality was observed (*p* for nonlinearity = 0.017), as shown in Figure 3.

### 3.3 Subgroup analysis

**Figure 4:**
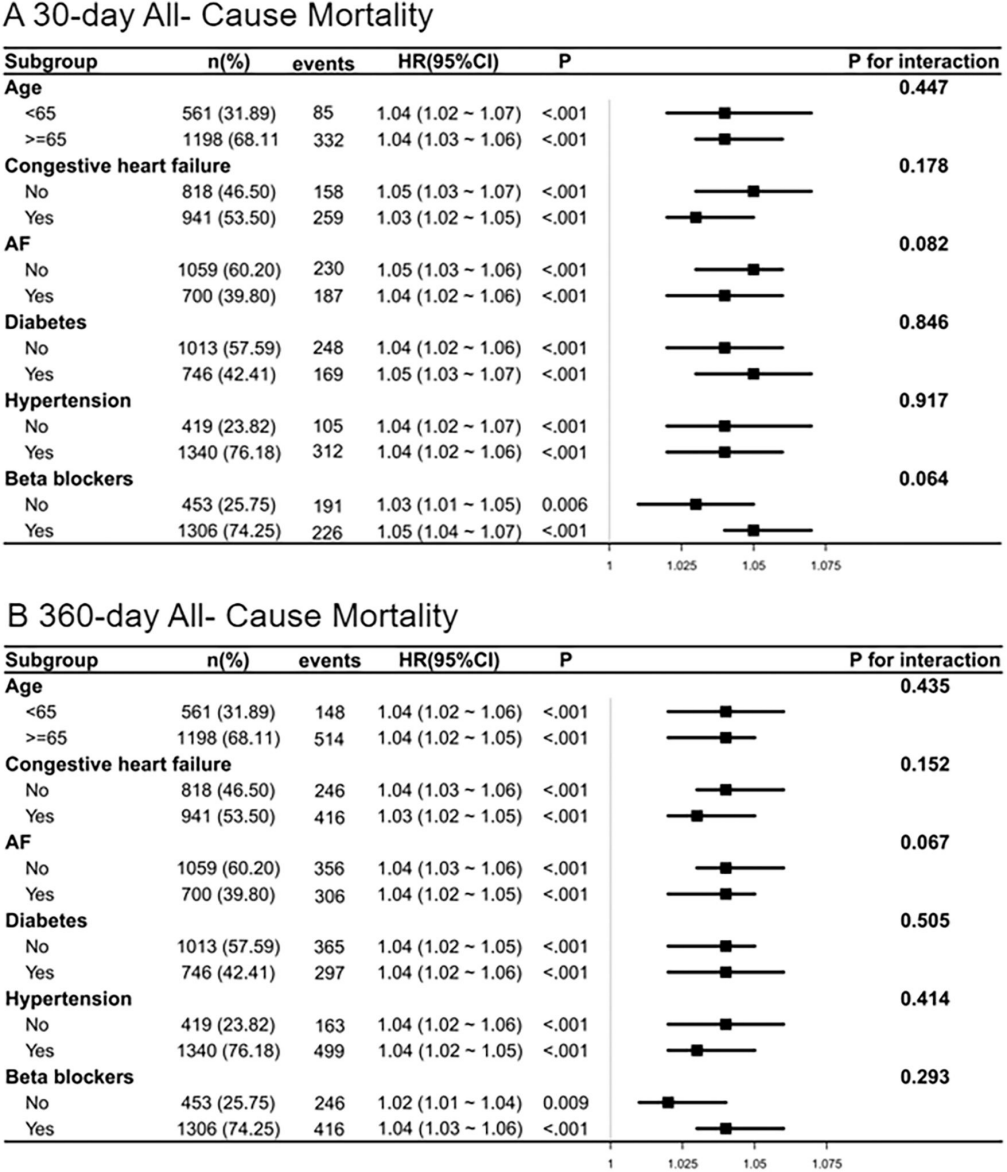
Subgroup analysis of the association between NPAR and all-cause mortality. **(A)** 30-day all-cause mortality; **(B)** 360-day all-cause mortality.

Subgroup analyses reinforced the stable link between higher NPAR values and elevated mortality risk across diverse clinical strata, as shown in Figure 4. As detailed in the figure, which includes patient counts and the number of mortality events for each stratum, a visual inspection of the forest plots for both 30-day and 360-day mortality reveals that the HR were directionally consistent across all examined subgroups. Furthermore, the 95% CI largely overlapped between strata for each variable. This suggests no substantial heterogeneity or evidence of significant effect modification by age, CHF, AF, diabetes, hypertension, or beta-blocker use. These results reinforce the utility of NPAR as a consistent and dependable prognostic marker for all-cause mortality among ICU-admitted MI patients, irrespective of their baseline clinical.

### 3.4 Feature selection

**Figure 5:**
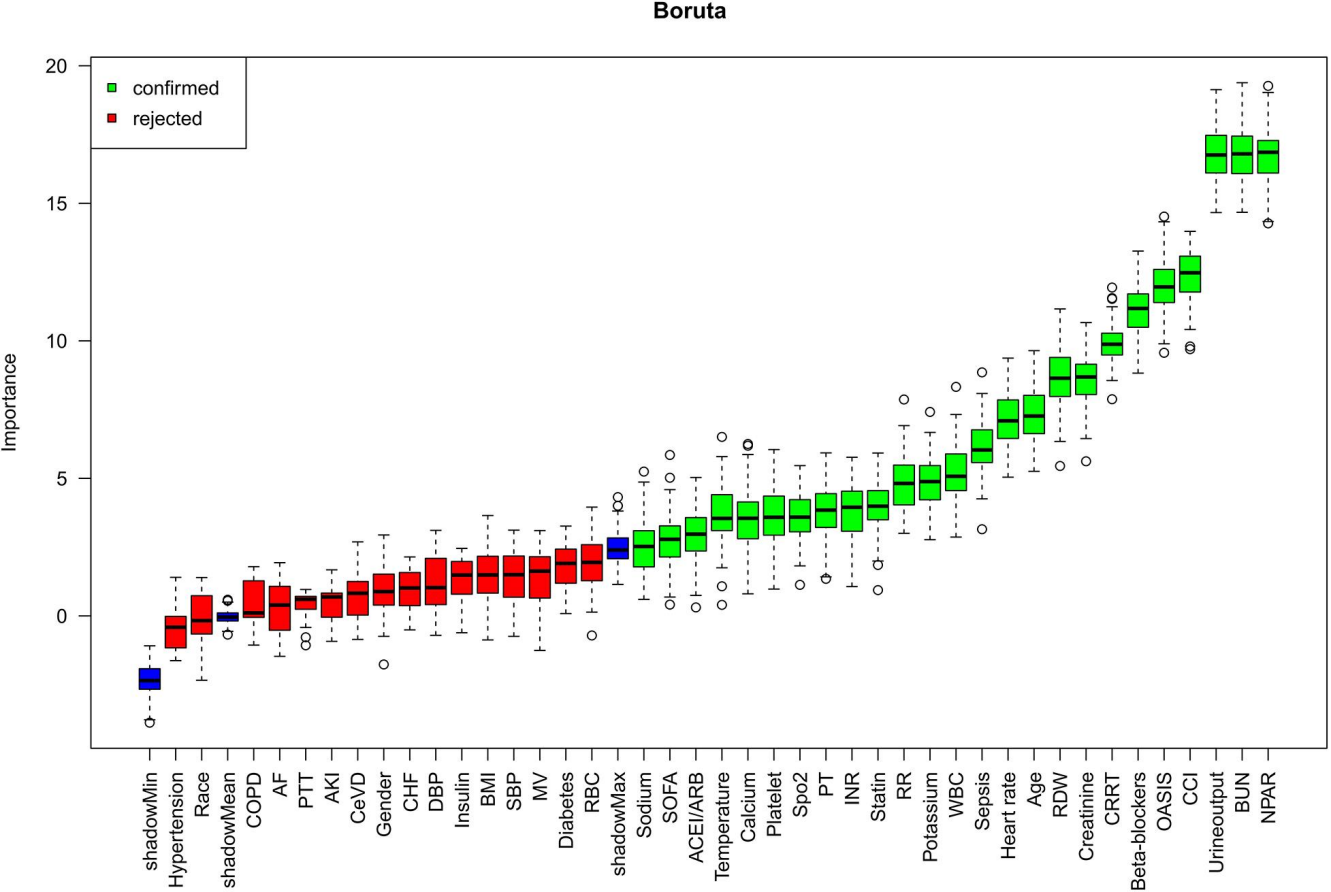
Feature selection based on the Boruta algorithm.

Figure 5 displays the outcomes of variable selection performed using the Boruta algorithm. Variables highlighted in green were identified as important predictors, while those in red were deemed unimportant. A total of 25 variables were ultimately selected as the most relevant predictors of all-cause mortality, ranked by descending Z-score importance as follows: NPAR, BUN, urine output, CCI, OASIS, Beta -blocker use, CRRT, creatinine, RDW, age, heart rate, sepsis, WBC, potassium, RR, statin use, INR, PT, SpO₂, platelets, calcium, temperature, ACEI/ARB, SOFA score, and sodium.

### 3.5 Development and evaluation of the mortality risk prediction model

**Figure 6:**
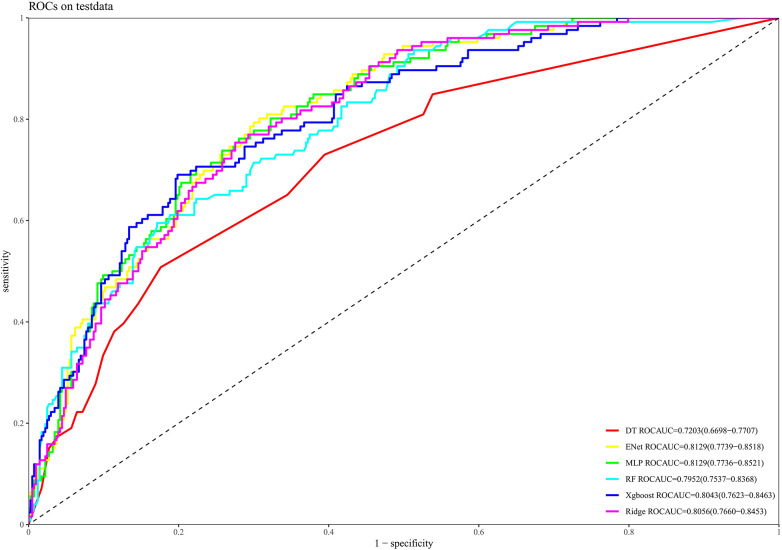
Receiver operating characteristic (ROC) curves of different machine learning models for predicting all-cause mortality on the test dataset.

Figure 6 presents the ROC curves for each machine learning algorithm, with model performance evaluated by the AUC. The AUC values were as follows: ENet = 0.8129, MLP = 0.8129, ridge regression = 0.8056, XGBoost = 0.8043, RF = 0.7952, and DT = 0.7203. Supplementary Figure S1 illustrates the calibration curves for each model evaluated on the test dataset. Overall, the calibration curves for ENet, RF, and XGBoost closely aligned with the reference line, indicating good consistency and calibration performance. As depicted in Supplementary Figure S2, decision curve analysis revealed that each model provided substantial yielded meaningful clinical value across a wide range of threshold probabilities, highlighting their promising applicability in real-world settings.

## 4 Discussion

In this large-scale retrospective cohort study, we established that an elevated admission NPAR is an independent predictor of both short- and long-term all-cause mortality in a broad cohort of critically ill MI patients. While previous studies have validated NPAR in various cardiovascular settings, our study provides several novel insights. We are the first to systematically confirm its prognostic utility in a large, heterogeneous, unselected ICU-based MI population, extending its relevance beyond more specific cohorts like general AMI or coronary care unit patients. Furthermore, by demonstrating its value not only in traditional Cox regression but also as a key feature in multiple machine learning models, our findings highlight NPAR's potential as a robust, easily accessible biomarker for integration into modern, data-driven risk stratification tools in this high-acuity clinical environment. Notably, the prognostic value of NPAR remained consistent across all examined subgroups, including patients with or without diabetes, hypertension, or atrial fibrillation. This suggests that NPAR reflects a core pathophysiological process involving systemic inflammation and nutritional status. Many common comorbidities, while important risk factors themselves, likely channel their detrimental effects through this central pathway of systemic stress that NPAR quantifies. From a clinical perspective, this consistency is a key strength. It validates NPAR as a robust and broadly applicable biomarker for the heterogeneous population of critically ill MI patients. Its utility is not diminished by these common comorbidities, which simplifies its potential integration into clinical practice as a universal indicator of systemic vulnerability.

### 4.1 NPAR and MI

NPAR is calculated by dividing the neutrophil percentage in peripheral blood by the serum albumin level, reflecting both systemic inflammation and nutritional status. It has also been established as an independent prognostic marker across various clinical conditions, including stroke, acute kidney injury, and septic shock (18–21). Recently, its application has extended into cardiovascular disease prognosis. For example, findings by Cai et al. indicated that higher NPAR levels were strongly linked to elevated risks of in-hospital and one-year mortality among patients in coronary care units. Their multivariate analysis further validated NPAR as a robust predictor of adverse clinical outcomes (22). Similarly, Lin et al. demonstrated that admission NPAR levels were independently correlated in relation to all-cause mortality at 180 and 365 days among patients diagnosed with AMI (23). These findings are consistent with another study involving critically ill individuals with coronary artery disease, which reported a strong positive link between elevated NPAR levels and higher all-cause mortality risk (24). Together, previous studies underscore the prognostic significance of NPAR across clinical contexts. Building on this foundation, the present analysis reinforces the link between NPAR and all-cause mortality in ICU-treated MI patients, validating its role as an independent predictor and emphasizing its potential utility in risk stratification. Unlike previous studies that mainly focused on general MI populations or coronary care unit settings, our study specifically investigated critically ill MI patients admitted to intensive care units. This ICU-specific focus advances existing NPAR literature by demonstrating its prognostic value in a more complex, high-acuity clinical environment. Furthermore, by incorporating NPAR into machine learning models, our study reveals improved predictive accuracy for short-term mortality, underscoring the marker's clinical utility in precision risk assessment.

Multiple pathophysiological processes may underlie the observed link between NPAR levels and overall mortality among individuals with MI. NPAR, derived from neutrophil percentage and albumin concentration, reflects the interplay between systemic inflammatory activity and nutritional status (25). Neutrophils serve as key mediators in post-MI inflammatory processes, playing pivotal roles in both tissue injury and subsequent repair mechanisms (26, 27). During MI, neutrophils migrate to necrotic myocardial tissue driven by pro-inflammatory signaling pathways (28). After infiltrating the infarcted myocardium, activated neutrophils intensify tissue damage by releasing large amounts of reactive oxygen species (ROS) and proteolytic enzymes, thereby facilitating adverse ventricular remodeling and contributing to increased mortality (29). In addition to producing ROS, activated neutrophils release proteolytic enzymes and inflammatory signaling molecules such as tumor necrosis factor-alpha (TNF-α), which further amplify inflammation, exacerbate tissue injury, and drive maladaptive cardiac remodeling (30).Conversely, serum albumin concentration has been independently linked to mortality risk in individuals with acute coronary syndrome (ACS) (31). Several studies have shown that lower serum albumin concentrations are associated with an increased risk of MI and higher mortality in individuals with ACS (32, 33). Serum albumin not only acts as a biomarker for nutritional status but also possesses significant antioxidant and anti-inflammatory properties. Albumin synthesis can be suppressed by inflammatory cytokines; for instance, Duran-Güell et al. demonstrated that serum albumin acts as an essential free radical scavenger, neutralizing free radicals and mitigating oxidative stress via multiple biochemical pathways (34). The role of albumin in combating oxidative stress and inflammation highlights its protective effect in various disease states, while lower serum albumin levels may indicate an imbalance of these protective mechanisms (35). The prognostic power of NPAR is likely rooted in its ability to capture a “dual-hit” state driving adverse post-MI remodeling. The elevated neutrophil component signifies an aggressive inflammatory attack, where neutrophils release matrix metalloproteinases (MMPs) degrade the myocardial matrix, leading to pathological fibrosis and adverse remodeling (36). Concurrently, the low albumin component indicates a compromised systemic defense with reduced antioxidant capacity (37), allowing this MMP-mediated damage to proceed unchecked. Thus, a high NPAR represents a state where an aggressive, MMP-driven assault on the myocardial matrix occurs simultaneously with a failure of the body's protective systems, a combination that directly promotes the adverse ventricular remodeling underlying post-MI heart failure. Thus, combining neutrophil proportion with serum albumin levels, NPAR offers a broader and more precise evaluation of a patient's overall risk status.

### 4.2 Clinical implications

NPAR, as a dual-dimensional biomarker integrating inflammatory response and nutritional status, exhibits distinct clinical value in prognostic assessment for MI patients admitted to the ICU. While established risk scores like GRACE and TIMI are invaluable for assessing ischemic and hemodynamic risk in MI patients, their focus is not on the host's systemic response. Our findings position NPAR as a powerful and accessible complement to these tools, not a replacement. Its clinical value lies in its ability to quantify a distinct pathophysiological axis: the inflammation-nutrition status, which is a critical determinant of outcomes in the ICU setting. By integrating these two dimensions into a single, cost-effective biomarker derived from routine blood tests, NPAR provides rapid, additive prognostic information. Its incremental value, therefore, likely comes from offering a window into the patient's systemic vulnerability, a dimension of risk not fully captured by traditional cardiac-focused scores. Incorporating NPAR into current risk stratification frameworks may aid clinicians in the timely recognition of high-risk patients, enabling earlier implementation of intensive monitoring or individualized therapies, including anti-inflammatory strategies and nutritional support.

### 4.3 Innovation and limitations

The principal innovation of this study, as detailed previously, is the validation of NPAR as a robust prognostic marker specifically within the complex, high-acuity ICU setting for MI patients, supported by both traditional statistical methods and machine learning models.

Despite its strengths, this study is subject to several limitations. First, its retrospective nature and reliance on data from a single-center source may lead to selection bias and limit the ability to draw causal conclusions. Second, although extensive covariate adjustment was performed in multivariate analyses, the potential impact of unmeasured confounders may still persist. Third, while our formal tests for interaction in the subgroup analyses were not statistically significant, we acknowledge that these tests may have been underpowered to detect modest or subtle effect modification. The non-significant *p*-values should therefore be interpreted with caution, as they do not definitively rule out the presence of interactions, particularly in smaller strata. Fourth, a key limitation is our inability to formally test the incremental prognostic value of NPAR when added to established scores like GRACE or TIMI. This was due to the absence of specific variables required for their calculation in the MIMIC-IV database. Future prospective studies are essential to determine whether NPAR improves risk reclassification beyond these conventional tools. Moreover, due to the ICD-based data extraction from the MIMIC-IV database does not preserve detailed subtype information and the absence of electrocardiogram data, troponin, BNP levels, and detailed MI subtype classification (e.g., STEMI and NSTEMI) in the database, we were unable to perform more refined diagnostic stratification and risk analysis. The lack of these MI-specific biomarkers and ECG findings may reduce the comprehensiveness and rigor of our prognostic model. The inability to differentiate STEMI from NSTEMI, in particular, limits the precision and external applicability of our findings. Future studies integrating biochemical and electrocardiographic indicators could further enhance the interpretability and predictive robustness of NPAR-based models. Finally, our evaluation of the machine learning models focused on the AUC, which assesses overall discrimination without requiring a pre-specified risk threshold. We did not report threshold-dependent metrics such as sensitivity and specificity, as the selection of an optimal cutoff was considered beyond the scope of this prognostic validation study. Future research is needed to establish a clinically meaningful threshold and validate the model's performance in a real-world decision-making context.

## 5 Conclusion

In conclusion, this retrospective study suggests that admission NPAR is an independent prognostic marker associated with both short- and long-term all-cause mortality among ICU-admitted MI patients. The inclusion of NPAR also enhanced the performance of machine learning models for predicting mortality. However, these findings should be interpreted within the context of the study's limitations, including the potential for residual confounding and the absence of key cardiac-specific data such as troponin and ECG findings. Therefore, while NPAR shows promise as an accessible and valuable biomarker for risk stratification, further validation in multi-center, prospective studies is warranted to confirm its clinical utility in the critical care management of MI patients.

## Data Availability

The original contributions presented in the study are included in the article/Supplementary Material, further inquiries can be directed to the corresponding author.
