# Supplementary material for: Prognostic value of the neutrophil percentage-to-albumin ratio for mortality in ICU patients with myocardial infarction: a retrospective cohort and machine learning analysis
Source: Front Cardiovasc Med. 2025 Dec 19;12:1631493. doi: 10.3389/fcvm.2025.1631493 (PMC12757425; doi:10.3389/fcvm.2025.1631493)
Supplement: Supplementary file 1 [file Table1.docx]

**Supplementary materials**

**Supplementary Tables**

**Supplementary Table 1** All Variables extracted from the MIMIC-IV Database.

| **Category** | **Variables Included** |
| --- | --- |
| Demographics | Age, Gender, Race, and BMI. |
| Vital parameters | Heart rate, RR, SBP, DBP, Temperature, and Spo2. |
| Severity scores | SOFA, OASIS, and CCI. |
| Underlying comorbid conditions | CHF, CeVD, COPD, AF, AKI, Sepsis, Diabetes, and Hypertension. |
| Laboratory indicators | RBC, WBC, Platelet, RDW, Neutrophils, Albumin, Sodium, Potassium, Calcium, BUN, Creatinine, INR, PT, PTT, and Urine output. |
| Medication usage | Statin, Insulin, Beta-blockers, and ACEI/ARB. |
| Clinical interventions | MV and CRRT. |
| Clinical outcomes | Hospital stay, Hospital Mortality, ICU stay, ICU Mortality, 30-day hospital Mortality, and 360-day hospital Mortality. |
| BMI, body mass index; SBP, systolic blood pressure; DBP, diastolic blood pressure; RR, respiratory rate; Spo2, pulse oxygen saturation; SOFA, sequential organ failure assessment; OASIS, Oxford Acute Severity of Illness Score; CCI, Charlson Comorbidity Index; CHF, congestive heart failure; CeVD, cerebrovascular disease; COPD, chronic obstructive pulmonary disease; AF, atrial fibrillation; AKI, acute kidney injury; RBC, red blood cell count; WBC, white blood cell count; RDW, red cell distribution width; BUN, blood urea nitrogen; INR, international normalized ratio; PT, prothrombin time; PTT, partial thromboplastin time; ACEI/ARB, angiotensin-converting enzyme inhibitor/angiotensin receptor blocker; MV, mechanical ventilation; CRRT, continuous renal replacement therapy. | |

**Supplementary Table 2** Missing data rates of clinical variables in ICU patients with myocardial infarction.

| **Variable** | **Missing Value Rate (%)** |
| --- | --- |
| Height | 26.20807277 |
| Weight | 15.40648096 |
| Calcium | 5.79874929 |
| Temperature | 3.75213189 |
| Urine output | 3.58158044 |
| INR | 2.33086981 |
| PT | 2.33086981 |
| PTT | 2.33086981 |
| RDW | 0.39795338 |
| Platelet | 0.28425242 |
| RBC | 0.17055145 |
| WBC | 0.17055145 |
| SBP | 0.11370097 |
| DBP | 0.11370097 |
| Sodium | 0.11370097 |
| Potassium | 0.11370097 |
| BUN | 0.11370097 |
| Heart rate | 0.05685048 |
| RR | 0.05685048 |
| Spo2 | 0.05685048 |
| SOFA | 0.05685048 |
| Creatinine | 0.05685048 |
| INR, international normalized ratio; PT, prothrombin time; PTT, partial thromboplastin time; RDW, red cell distribution width; RBC, red blood cell count; WBC, white blood cell count; SBP, systolic blood pressure; DBP, diastolic blood pressure; BUN, blood urea nitrogen; RR, respiratory rate; Spo2, pulse oxygen saturation; SOFA, sequential organ failure assessment | |

**Supplementary Table 3 Variance Inflation Factor (VIF) Results for Variables**

| Variables | VIF |
| --- | --- |
| Age | 2.04 |
| Gender | 1.14 |
| Race | 1.06 |
| BMI | 1.17 |
| Temperature | 1.17 |
| Heart Rate | 1.40 |
| SBP | 1.58 |
| DBP | 1.73 |
| RR | 1.22 |
| Spo2 | 1.16 |
| SOFA | 1.38 |
| OASIS | 1.67 |
| CCI | 2.92 |
| CHF | 1.30 |
| CeVD | 1.18 |
| COPD | 1.17 |
| AF | 1.18 |
| AKI | 1.19 |
| Sepsis | 1.37 |
| Diabetes | 1.66 |
| Hypertension | 1.19 |
| RBC | 1.41 |
| WBC | 1.09 |
| Platelet | 1.24 |
| RDW | 1.36 |
| Neutrophils | 1.13 |
| Albumin | 1.69 |
| Sodium | 1.14 |
| Potassium | 1.25 |
| Calcium | 1.32 |
| BUN | 2.11 |
| Creatinine | 2.17 |
| INR | 3.34 |
| PT | 3.85 |
| PTT | 1.09 |
| Urine output | 1.42 |
| Statin | 1.29 |
| Insulin | 1.54 |
| Beta blockers | 1.23 |
| ACEI/ARB | 1.11 |
| MV | 1.13 |
| CRRT | 1.42 |
| BMI, body mass index; SBP, systolic blood pressure; DBP, diastolic blood pressure; RR, respiratory rate; Spo2, pulse oxygen saturation; SOFA, sequential organ failure assessment; OASIS, Oxford Acute Severity of Illness Score; CCI, Charlson Comorbidity Index; CHF, congestive heart failure; CeVD, cerebrovascular disease; COPD, chronic obstructive pulmonary disease; AF, atrial fibrillation; AKI, acute kidney injury; RBC, red blood cell count; WBC, white blood cell count; RDW, red cell distribution width; BUN, blood urea nitrogen; INR, international normalized ratio; PT, prothrombin time; PTT, partial thromboplastin time; NPAR, neutrophil percentage-to-albumin ratio; ACEI/ARB, angiotensin-converting enzyme inhibitor/angiotensin receptor blocker; MV, mechanical ventilation; CRRT, continuous renal replacement therapy. | |

**Supplementary Table 4** Univariate Cox regression analysis of risk factors for all-cause mortality in ICU patients with myocardial infarction.

| **Variables** | **P** | **HR (95%CI)** |
| --- | --- | --- |
| Gender |  |  |
| Female |  | 1.00 (Reference) |
| Male | <.001 | 0.70 (0.58 ~ 0.85) |
| Race |  |  |
| Other |  | 1.00 (Reference) |
| White | 0.162 | 0.87 (0.71 ~ 1.06) |
| Age | <.001 | 1.03 (1.02 ~ 1.04) |
| BMI | 0.052 | 0.99 (0.97 ~ 1.00) |
| Heart rate | <.001 | 1.01 (1.01 ~ 1.02) |
| RR | <.001 | 1.05 (1.03 ~ 1.06) |
| Spo2 | <.001 | 0.96 (0.94 ~ 0.97) |
| OASIS | <.001 | 1.07 (1.06 ~ 1.08) |
| CCI | <.001 | 1.18 (1.15 ~ 1.22) |
| CHF |  |  |
| No |  | 1.00 (Reference) |
| Yes | <.001 | 1.50 (1.23 ~ 1.82) |
| COPD |  |  |
| No |  | 1.00 (Reference) |
| Yes | <.001 | 1.44 (1.17 ~ 1.77) |
| AF |  |  |
| No |  | 1.00 (Reference) |
| Yes | 0.028 | 1.24 (1.02 ~ 1.51) |
| AKI |  |  |
| No |  | 1.00 (Reference) |
| Yes | <.001 | 2.53 (1.75 ~ 3.67) |
| Sepsis |  |  |
| No |  | 1.00 (Reference) |
| Yes | <.001 | 3.93 (3.02 ~ 5.11) |
| Hypertension |  |  |
| No |  | 1.00 (Reference) |
| Yes | 0.482 | 0.92 (0.74 ~ 1.15) |
| RBC | 0.011 | 0.86 (0.77 ~ 0.97) |
| WBC | 0.008 | 1.01 (1.01 ~ 1.01) |
| Platelet | 0.894 | 1.00 (1.00 ~ 1.00) |
| RDW | <.001 | 1.16 (1.13 ~ 1.19) |
| Calcium | 0.227 | 0.93 (0.83 ~ 1.05) |
| BUN | <.001 | 1.02 (1.01 ~ 1.02) |
| Creatinine | <.001 | 1.14 (1.10 ~ 1.19) |
| INR | <.001 | 1.12 (1.06 ~ 1.19) |
| PT | <.001 | 1.01 (1.01 ~ 1.02) |
| Urine output | <.001 | 0.99 (0.99 ~ 0.99) |
| Statin |  |  |
| No |  | 1.00 (Reference) |
| Yes | <.001 | 0.46 (0.38 ~ 0.56) |
| Insulin |  |  |
| No |  | 1.00 (Reference) |
| Yes | 0.067 | 0.83 (0.68 ~ 1.01) |
| Beta-blockers |  |  |
| No |  | 1.00 (Reference) |
| Yes | <.001 | 0.33 (0.27 ~ 0.40) |
| ACEI/ARB |  |  |
| No |  | 1.00 (Reference) |
| Yes | <.001 | 0.52 (0.41 ~ 0.67) |
| CRRT |  |  |
| No |  | 1.00 (Reference) |
| Yes | <.001 | 2.88 (2.29 ~ 3.62) |
| BMI, body mass index; RR, respiratory rate; Spo2, pulse oxygen saturation; OASIS, Oxford Acute Severity of Illness Score; CCI, Charlson Comorbidity Index; CHF, congestive heart failure; COPD, chronic obstructive pulmonary disease; AF, atrial fibrillation; AKI, acute kidney injury; RBC, red blood cell count; WBC, white blood cell count; RDW, red cell distribution width; BUN, blood urea nitrogen; INR, international normalized ratio; PT, prothrombin time; ACEI/ARB, angiotensin-converting enzyme inhibitor/angiotensin receptor blocker; CRRT, continuous renal replacement therapy. | | |

**Supplementary Table 5 Proportional Hazards Assumption Test Results for the Cox Model**

| Variable | Chi-Square | df | p-value |
| --- | --- | --- | --- |
| NPAR | 0.5 | 1 | 0.48 |
| Age | 0.37 | 1 | 0.54 |
| Gender | 0.03 | 1 | 0.86 |
| Race | 1.61 | 1 | 0.2 |
| Heart Rate | 1.1 | 1 | 0.29 |
| SBP | 0.33 | 1 | 0.57 |
| SPo2 | < 0.01 | 1 | 0.98 |
| CHF | 1.35 | 1 | 0.24 |
| AF | 0.06 | 1 | 0.8 |
| RDW | 1.18 | 1 | 0.27 |
| BUN | 1.51 | 1 | 0.22 |
| PT | 0.26 | 1 | 0.61 |
| Urine output | 3.58 | 1 | 0.55 |
| Beta blockers | 0.58 | 1 | 0.45 |
| GLOBAL | 20.2 | 14 | 0.12 |
| NPAR, Neutrophil Percentage-to-Albumin Ratio; df, degrees of freedom; SBP, systolic blood pressure; SpO2, pulse oxygen saturation; CHF, congestive heart failure; AF, atrial fibrillation; RDW, red cell distribution width; BUN, blood urea nitrogen; PT, prothrombin time; GLOBAL, Global Test. | | | |

**Supplementary Figures**

**Supplementary Figure 1**


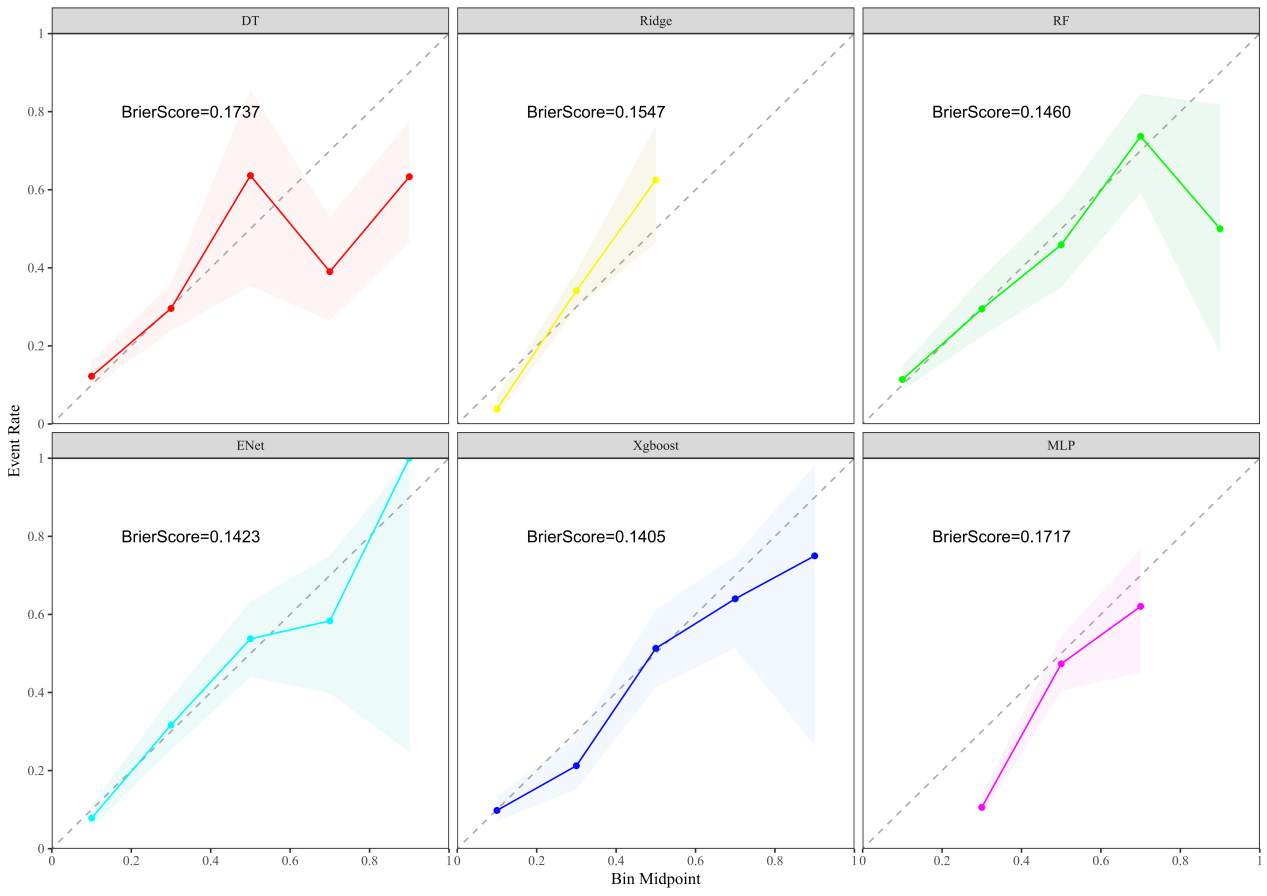
**Supplementary Figure 1** Calibration curves of different machine learning models for predicting all-cause mortality.

**Supplementary Figure 2**


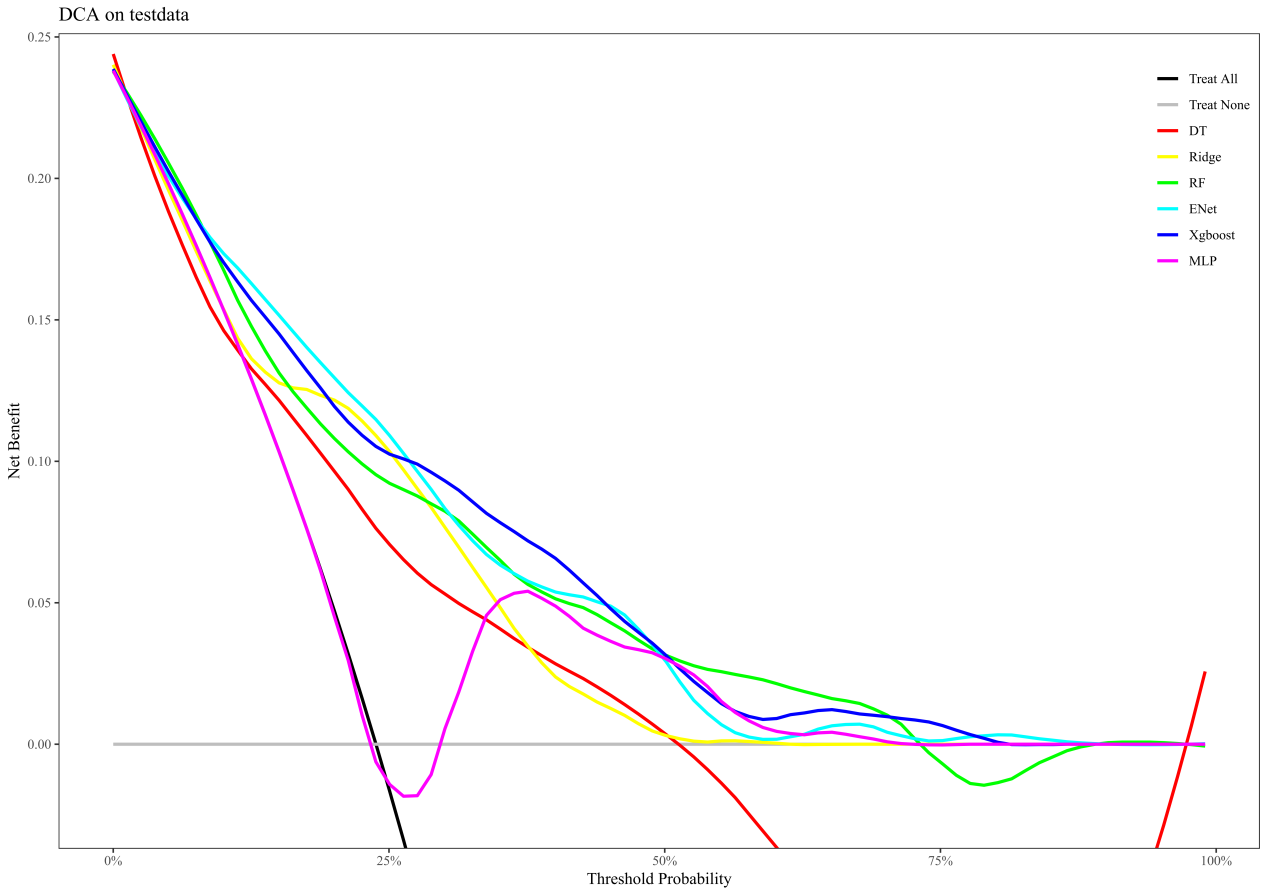
**Supplementary Figure 2** Decision curve analysis (DCA) of different machine learning models for predicting all-cause mortality.
